# Supplementary material for: Online Resources for People Who Self-Harm and Those Involved in Their Informal and Formal Care: Observational Study with Content Analysis
Source: Int J Environ Res Public Health. 2020 May 18;17(10):3532. doi: 10.3390/ijerph17103532 (PMC7277667; doi:10.3390/ijerph17103532)
Supplement: Supplementary file 1 [file ijerph-17-03532-s001.pdf]

International Journal of
Environmental Research and Public Health

MDPI

Supplementary A. The 49 Websites identified by the scoping work.

#	Website Name/Organisation	Link
1	National Institute for Health and Care Excellence	https://www.nice.org.uk/
2	British Medical Journal Best Practice	https://bestpractice.bmj.com/info/
3	National Health Service	https://www.nhs.uk/
4	Patient	https://patient.info/
5	Royal College of Psychiatrists	https://www.rcpsych.ac.uk/
6	Royal College of Paediatrics and Child Health	https://www.rcpch.ac.uk/
7	Royal College of General Practitioners	https://www.rcgp.org.uk/
8	Royal College of Nursing	https://www.rcn.org.uk/
9	World Health Organisation	https://www.who.int/
10	Public Health England	https://www.gov.uk/government/organisations/public-health-england
11	Public Health Scotland	https://www.gov.scot/
12	Public Health Wales	http://www.wales.nhs.uk/sitesplus/888/home
13	HSC Public Health Agency	https://www.publichealth.hscni.net/
14	Royal Society for Public Health	https://www.rsph.org.uk/
15	Centre for Mental Health	https://www.centreformentalhealth.org.uk/
16	British Association for Behavioural and Cognitive Psychotherapies	https://www.babcp.com/Default.aspx
17	British Association for Counselling and Psychotherapy	https://www.bacp.co.uk/
18	UK Council for Psychotherapy	https://www.psychotherapy.org.uk/
19	British Psychological Society	https://www.bps.org.uk/
20	Samaritans	https://www.samaritans.org/
21	SANE	http://www.sane.org.uk/home
22	CALM	https://www.thecalmzone.net/
23	Childline	https://www.childline.org.uk/
24	SelfHarmUK	https://www.selfharm.co.uk/
25	LifeSIGNS	http://www.lifesigns.org.uk/
26	Harmless	http://www.harmless.org.uk/
27	Recover Your Life	http://www.recoveryourlife.com/
28	Inspire Wellbeing	https://www.inspirewellbeing.org/
29	Papyrus	https://papyrus-uk.org/
30	BEAT	https://www.beateatingdisorders.org.uk/
31	Rethink Mental Illness	https://www.rethink.org
32	Mind	https://www.mind.org.uk/
33	Battle Scars	https://www.battle-scars-self-harm.org.uk/
34	Mental Health Matters	https://www.mhm.org.uk/
35	Youth Access	https://www.youthaccess.org.uk/
36	Mood Swings	http://www.moodswings.org.uk/
37	No Panic	https://www.nopanic.org.uk/
38	Life Centre	https://lifecentre.uk.com/
39	Survivors UK	https://www.survivorsuk.org/
40	Self Injury Support Network	https://www.selfinjurysupport.org.uk/
41	The Mix	https://www.themix.org.uk/
42	Young Minds	https://youngminds.org.uk/
43	MindEd	https://www.minded.org.uk/
44	Child Protection Company	https://www.childprotectioncompany.com/CPC/index.html
45	The Silver Line	https://www.thesilverline.org.uk/
46	Adullam Ministries	http://www.adullam-ministries.org.uk/site/
47	Scottish Association for Mental Health	https://www.samh.org.uk/

48	Action Mental Health Northern Ireland	https://www.amh.org.uk/
49	The King's Fund	https://www.kingsfund.org.uk/

Supplementary B. The search strategy and results for each website.

1. National Institute for Health and Care Excellence

Link: https://www.nice.org.uk/

Date searched: 16/08/19

Search strategy:

- Website browsed
- Individually searched "self harm", "self injury", "deliberate self harm", "parasuicide", "self poisoning", "self cutting" and "self injurious behaviour" in main search bar, filtered to show only advice and guidance

Total hits = 82, of which 16 were duplicates

Results which met eligibility criteria for full-text inclusion:

#	Webpage Name	Link
1	Psychotropic medicines in people with learning disabilities whose behaviour challenges	https://www.nice.org.uk/advice/ktt19
2	Self-harm in over 8s: short-term management and prevention of recurrence	https://www.nice.org.uk/guidance/cg16
3	Self-harm in over 8s: long-term management	https://www.nice.org.uk/guidance/cg133
4	Self-harm (quality standard)	https://www.nice.org.uk/guidance/qs34
5	Self-harm in over 8s: short- and long-term management and prevention of recurrence	https://www.nice.org.uk/guidance/indevelopment/gid-ng10148
6	Borderline personality disorder: recognition and management	https://www.nice.org.uk/guidance/cg78
7	Mental health of adults in contact with the criminal justice system	https://www.nice.org.uk/guidance/ng66
8	Mental health of adults in contact with the criminal justice system	https://www.nice.org.uk/guidance/qs163
9	Obsessive-compulsive disorder and body dysmorphic disorder: treatment	https://www.nice.org.uk/guidance/cg31
10	Depression in children and young people: identification and management	https://www.nice.org.uk/guidance/ng134
11	Generalised anxiety disorder and panic disorder in adults: management	https://www.nice.org.uk/guidance/cg113
12	Bipolar disorder: assessment and management	https://www.nice.org.uk/guidance/cg185
13	Challenging behaviour and learning disabilities: prevention and interventions for people with learning disabilities whose behaviour challenges	https://www.nice.org.uk/guidance/ng11
14	Psychosis and schizophrenia in children and young people: recognition and management	https://www.nice.org.uk/guidance/cg155
15	Social anxiety disorder: recognition, assessment and treatment (CG159)	https://www.nice.org.uk/guidance/cg159
16	Personality disorders: borderline and antisocial	https://www.nice.org.uk/guidance/qs88
17	Antenatal and postnatal mental health	https://www.nice.org.uk/guidance/qs115
18	Autism spectrum disorder in under 19s: support and management	https://www.nice.org.uk/guidance/cg170
19	Autism spectrum disorder in under 19s: recognition, referral and diagnosis	https://www.nice.org.uk/guidance/cg128
20	Depression in adults: recognition and management	https://www.nice.org.uk/guidance/cg90
21	Eating disorders: recognition and treatment	https://www.nice.org.uk/guidance/ng69
22	Bipolar disorder in adults	https://www.nice.org.uk/guidance/qs95

23	Depression in children and young people	https://www.nice.org.uk/guidance/qs48
24	Psychosis and schizophrenia in adults: prevention and management	https://www.nice.org.uk/guidance/cg178
25	Bipolar disorder, psychosis and schizophrenia in children and young people	https://www.nice.org.uk/guidance/qs102
26	Eating disorders	https://www.nice.org.uk/guidance/qs175
27	Anxiety disorders	https://www.nice.org.uk/guidance/qs53
28	Antisocial behaviour and conduct disorders in children and young people: recognition and management	https://www.nice.org.uk/guidance/cg158
29	Autism spectrum disorder in adults: diagnosis and management	https://www.nice.org.uk/guidance/cg142
30	Antisocial behaviour and conduct disorders in children and young people	https://www.nice.org.uk/guidance/qs59
31	Common mental health problems: identification and pathways to care	https://www.nice.org.uk/guidance/cg123
32	Depression in adults with a chronic physical health problems: recognition and management	https://www.nice.org.uk/guidance/cg91
33	Learning disability: behaviour that challenges	https://www.nice.org.uk/guidance/qs101

2. British Medical Journal Best Practice

Link: https://bestpractice.bmj.com/info/

Date searched: 20/8/19

Search strategy:

- Website browsed
- Search bar not used as required log-in

Total hits = 0

3. National Health Service

Link: https://www.nhs.uk/

Date searched: 19/08/19

Search strategy:

- Website browsed
- Individually searched “self harm”, “self injury”, “deliberate self harm”, “parasuicide”, “self poisoning”, “self cutting” and “self injurious behaviour” in main search bar

Total hits = 224, of which 4 were duplicates

Results which met eligibility criteria for full-text inclusion:

#	**Webpage Name**	**Link**
1	Self harm: overview	https://www.nhs.uk/conditions/self-harm/
2	Calm harm	https://www.nhs.uk/apps-library/calm-harm/
3	Talking about self harm	https://www.nhs.uk/video/Pages/Self-harm.aspx
4	Study looks at self-harm in young people	https://www.nhs.uk/news/mental-health/study-looks-at-self-harm-in-young-people/
5	Dealing with a mental health crisis or emergency	https://www.nhs.uk/using-the-nhs/nhs-services/mental-health-services/dealing-with-a-mental-health-crisis-or-emergency/
6	Distract	https://www.nhs.uk/apps-library/distract/
7	Depression and low mood	https://www.nhs.uk/video/Pages/bsl-depression.aspx
8	BlueIce	https://www.nhs.uk/apps-library/blueice/
9	Bullied children more likely to self-harm	https://www.nhs.uk/news/mental-health/bullied-children-more-likely-to-self-harm/

10	Goth teens are increased risk of depression and self harm	https://www.nhs.uk/news/mental-health/goth-teens-at-increased-risk-of-depression-and-self-harm/
11	Therapy reduces risk of suicide or self-harm	https://www.nhs.uk/news/mental-health/therapy-reduces-risk-of-suicide-or-self-harm/
12	ChatHealth	https://www.nhs.uk/apps-library/chathealth/
13	Nearly a quarter of 14-year-old girls in UK self-harming, charity reports	https://www.nhs.uk/news/mental-health/nearly-quarter-14-year-old-girls-uk-self-harming-charity-reports/
14	Treatment of depression in children needs improving	https://www.nhs.uk/news/mental-health/treatment-of-depression-in-children-needs-improving/
15	Worrying rise in reports of self-harm among teenage girls in UK	https://www.nhs.uk/news/mental-health/worrying-rise-reports-self-harm-among-teenage-girls-uk/
16	Weight loss surgery linked to increased self-harm risk	https://www.nhs.uk/news/mental-health/weight-loss-surgery-linked-to-increased-self-harm-risk/

4. Patient

Link: https://patient.info/

Date searched: 18/9/19

Search strategy:

- Website browsed
- Individually searched “self harm”, “self injury”, “deliberate self harm”, “self injurious behaviour”, “self poisoning”, “self cutting”, “parasuicide” and “deliberate self harm” in main search bar, filtered to show only ‘conditions’ and ‘features’

Total hits = 3060

Due to high volume of results and decreasing relevance, only the first 100 search results for each search term were screened

Modified hits = 600, of which 256 were duplicates

Results which met eligibility criteria for full-text inclusion:

#	Webpage Name	Link
1	Self-harm in young people	https://patient.info/childrens-health/surviving-adolescence/self-harm-in-young-people
2	What to do if you get the urge to self-harm	https://patient.info/news-and-features/what-to-do-if-you-get-the-urge-to-self-harm
3	Paracetamol overdose	https://patient.info/treatment-medication/paracetamol-overdose
4	How to improve your self-esteem and be kinder to yourself	https://patient.info/news-and-features/how-to-boost-self-esteem
5	How to practise self-care	https://patient.info/news-and-features/what-is-self-care
6	Cognitive behavioural therapy	https://patient.info/mental-health/cognitive-behavioural-therapy-cbt-leaflet
7	Dealing with depression	https://patient.info/mental-health/depression-leaflet/dealing-with-depression
8	Depression	https://patient.info/mental-health/depression-leaflet
9	Mindfulness	https://patient.info/mental-health/stress-management/mindfulness

5. Royal College of Psychiatrists

Link: https://www.rcpsych.ac.uk/

Date searched: 19/8/19

Search strategy:

- Website browsed
- Individually searched "self harm", "self injury", "deliberate self harm", "parasuicide", "self poisoning", "self cutting" and "self injurious behaviour" in main search bar

Total hits = 133, of which 33 were duplicates

Results which met eligibility criteria for full-text inclusion:

#	Webpage Name	Link
1	Self-harm	https://www.rcpsych.ac.uk/mental-health/problems-disorders/self-harm?searchTerms=%22self%20harm%22
2	Self-Harm and Suicide Prevention Competence Frameworks	https://www.rcpsych.ac.uk/improving-care/nccmh/other-work/self-harm-and-suicide-prevention-competence-frameworks?searchTerms=%22self%20harm%22<br>https://www.rcpsych.ac.uk/docs/default-source/improving-care/nccmh/nccmh-self-harm-and-suicide-prevention-competence-framework-children-and-young.pdf?sfvrsn=29d0a351_2<br>https://www.rcpsych.ac.uk/docs/default-source/improving-care/nccmh/nccmh-self-harm-and-suicide-prevention-competence-framework-adults-and-older-adults.pdf?sfvrsn=5c12349f_2<br>https://www.rcpsych.ac.uk/docs/default-source/improving-care/nccmh/nccmh-self-harm-and-suicide-prevention-competence-framework-public-health.pdf?sfvrsn=341fb3cd_2<br>https://www.rcpsych.ac.uk/docs/default-source/improving-care/nccmh/nccmh-self-harm-and-suicide-prevention-competence-framework-service-user-and-carer-report.pdf?sfvrsn=959b6695_2
3	Self-harm in young people: for parents and carers	https://www.rcpsych.ac.uk/mental-health/parents-and-young-people/information-for-parents-and-carers/self-harm-in-young-people-for-parents-and-carers?searchTerms="self%20harm"
4	Position Statement 2010: Self-harm, suicide and risk: a summary	https://www.rcpsych.ac.uk/docs/default-source/improving-care/better-mh-policy/position-statements/ps03-2010.pdf?sfvrsn=aece6c9c_4
5	Suicide Prevention National Transformation Programme	https://www.rcpsych.ac.uk/improving-care/nccmh/national-suicide-prevention-programme?searchTerms=%22self%20harm%22<br>https://www.rcpsych.ac.uk/docs/default-source/improving-care/nccmh/suicide-prevention/coping-with-self-harm-brochure.pdf?sfvrsn=336b1409_2<br>https://www.rcpsych.ac.uk/docs/default-source/improving-care/nccmh/suicide-prevention/young-people-who-self-harm-a-guide-for-school-staff.pdf?sfvrsn=e6ebf7ca_2
6	Special Interest Days – Suicide and Self-harm – January 2017	https://www.rcpsych.ac.uk/improving-care/ccqi/quality-networks-accreditation/psychiatric-liaison-accreditation-network-plan/events/special-interest-days?searchTerms=%22self%20harm%22
7	Feeling on the edge	https://www.rcpsych.ac.uk/mental-health/problems-disorders/feeling-on-the-edge?searchTerms=%22self%20harm%22
8	Feeling overwhelmed	https://www.rcpsych.ac.uk/mental-health/problems-disorders/feeling-overwhelmed?searchTerms=%22self%20harm%22
9	Psychiatrists should consider impact of social media on all children they assess, leading medical body says for first time	https://www.rcpsych.ac.uk/news-and-features/latest-news/detail/2019/03/30/psychiatrists-should-consider-impact-of-social-media-on-all-children-they-assess-leading-medical-body-says-for-first-time?searchTerms=%22self%20harm%22
10	U can cope! How to cope when life is difficult – for young people	https://www.rcpsych.ac.uk/mental-health/parents-and-young-people/young-people/u-can-cope!-how-to-cope-when-life-is-difficult-for-young-people?searchTerms=%22self%20harm%22

11	Personality disorder	https://www.rcpsych.ac.uk/mental-health/problems-disorders/personality-disorder?searchTerms=%22self%20harm%22
12	Depression in young people – helping children to cope – for parents and carers	https://www.rcpsych.ac.uk/mental-health/parents-and-young-people/information-for-parents-and-carers/depression-in-young-people---helping-children-to-cope-for-parents-and-carers?searchTerms=%22self%20harm%22
13	Psychosis – for young people	https://www.rcpsych.ac.uk/mental-health/parents-and-young-people/young-people/psychosis---for-young-people?searchTerms=%22self%20harm%22
14	Bipolar disorder: for young people	https://www.rcpsych.ac.uk/mental-health/parents-and-young-people/young-people/bipolar-disorder-for-young-people?searchTerms=%22self%20harm%22
15	Alcohol and depression	https://www.rcpsych.ac.uk/mental-health/problems-disorders/alcohol-and-depression?searchTerms=%22self%20harm%22
16	Schizophrenia for parents and carers	https://www.rcpsych.ac.uk/mental-health/parents-and-young-people/information-for-parents-and-carers/schizophrenia-for-parents-and-carers?searchTerms=%22self%20harm%22
17	Schizophrenia	https://www.rcpsych.ac.uk/mental-health/problems-disorders/schizophrenia?searchTerms=%22self%20harm%22
18	Bipolar disorder information for parents and carers	https://www.rcpsych.ac.uk/mental-health/parents-and-young-people/information-for-parents-and-carers/bipolar-affective-disorder-for-parents-carers-and-anyone-who-works-with-young-people?searchTerms=%22self%20harm%22
19	Schizoaffective disorder	https://www.rcpsych.ac.uk/mental-health/problems-disorders/schizoaffective-disorder?searchTerms=%22self%20harm%22
20	Eating well and mental health	https://www.rcpsych.ac.uk/mental-health/problems-disorders/eating-well-and-mental-health?searchTerms=%22self%20harm%22
21	Mental health in pregnancy	https://www.rcpsych.ac.uk/mental-health/treatments-and-wellbeing/mental-health-in-pregnancy?searchTerms=%22self%20harm%22
22	The child with general learning disability: for parents and carers	https://www.rcpsych.ac.uk/mental-health/parents-and-young-people/information-for-parents-and-carers/the-child-with-general-learning-disability-for-parents-and-carers?searchTerms=%22self%20injury%22

6. Royal College of Paediatrics and Child Health

Link: https://www.rcpch.ac.uk/
Date searched: 21/8/19
Search strategy:

- Website browsed
- Individually searched "self harm", "self injury", "deliberate self harm", "parasuicide", "self poisoning", "self cutting" and "self injurious behaviour" in main search bar

Total hits = 2, of which 0 were duplicates
No results met eligibility criteria for full-text inclusion

7. Royal College of General Practitioners

Link: https://www.rcgp.org.uk/
Date searched: 20/8/19
Search strategy:

- Website browsed
- Individually searched "self harm", "self injury", "self poisoning", "deliberate self harm", "parasuicide", "self cutting", "self injurious behaviour" in main search bar

Total hits = 15, of which 4 were duplicates

Results which met eligibility criteria for full-text inclusion:

#	Webpage Name	Link
1	Mental health: self-harming in older adults has distinct characteristics	https://www.rcgp.org.uk/clinical-and-research/about/clinical-news/2019/may/mental-health-self-harming-in-older-adults-has-distinct-characteristics.aspx
2	Youth mental health resources	https://www.rcgp.org.uk/clinical-and-research/resources/a-to-z-clinical-resources/youth-mental-health/youth-mental-health-resources.aspx<br>https://www.rcgp.org.uk/clinical-and-research/resources/a-to-z-clinical-resources/youth-mental-health/youth-mental-health-resources.aspx<br>https://www.rcgp.org.uk/-/media/Files/CIRC/Child-and-Adolescent-Health/RCGP-The-Daily-Mail-News-Article-December-2014.ashx?la=en
3	Youth mental health policies and reports	https://www.rcgp.org.uk/clinical-and-research/resources/a-to-z-clinical-resources/youth-mental-health/youth-mental-health-policies-and-reports.aspx<br>https://assets.publishing.service.gov.uk/government/uploads/system/uploads/attachment_data/file/252660/33571_2901304_CMO_Chapter_10.pdf<br>https://assets.publishing.service.gov.uk/government/uploads/system/uploads/attachment_data/file/216853/CYP-Mental-Health.pdf
4	Mental health	https://www.rcgp.org.uk/clinical-and-research/resources/a-to-z-clinical-resources/mental-health.aspx<br>https://www.rcgp.org.uk/clinical-and-research/resources/toolkits/mental-health-toolkit.aspx<br>https://www.rcgp.org.uk/-/media/Files/CIRC/Mental-Health---2014/Mental-Health-2017/RCGP-PS-mental-health-nov-2017.ashx?la=en<br>https://www.sabp.nhs.uk/moodhive<br>https://www.rcgp.org.uk/clinical-and-research/resources/toolkits/-/media/BEA12970F05A4BC48DC64CD59474188B.ashx<br>https://www.rcgp.org.uk/clinical-and-research/resources/toolkits/~/media/64FB9338D39B494FA635B9A5C3A8B424.ashx<br>https://www.rcgp.org.uk/clinical-and-research/resources/toolkits/~/media/0309F0E94E5D4EA8B6414E3BF637971F.ashx<br>https://www.rcgp.org.uk/clinical-and-research/resources/toolkits/-/media/54C328D6A0434A4982338AC03FF424BF.ashx<br>https://www.rcgp.org.uk/clinical-and-research/resources/toolkits/~/media/97B6C76D1B1F4FA7924B7DBD2044AEF1.ashx<br>https://www.mentalhealth.org.uk/sites/default/files/fundamental-facts-about-mental-health-2016.pdf
5	The state of youth mental health	https://www.rcgp.org.uk/clinical-and-research/resources/a-to-z-clinical-resources/youth-mental-health/the-state-of-youth-mental-health.aspx
6	Impact on the physical health of people living with a mental illness	https://www.rcgp.org.uk/clinical-and-research/about/clinical-news/2018/april/impact-on-the-physical-health-of-people-living-with-a-mental-illness.aspx

8. Royal College of Nursing

Link: https://www.rcn.org.uk/

Date searched: 20/8/19

Search strategy:

- Website browsed
- Individually searched "self harm", "self injury", "self poisoning", "deliberate self harm", "parasuicide", "self cutting" and "self injurious behaviour" in main search bar

Total hits = 4, of which 0 were duplicates

Results which met eligibility criteria for full-text inclusion:

#	Webpage Name	Link
1	Children and Young People: Mental Health Promotion	https://www.rcn.org.uk/library/subject-guides/children-and-young-people-mental-health-promotion#tab1 https://www.rcn.org.uk/professional-development/publications/pub-006021 https://www.rcn.org.uk/professional-development/publications/pub-004587 https://www.rcn.org.uk/professional-development/publications/pub-003311

9. World Health Organisation

Link: https://www.who.int/

Date searched: 18/9/19

Search strategy:

- Website browsed
- Individually searched "self harm", "parasuicide", "self injury", "self cutting", "self poisoning", "self injurious behaviour" and "deliberate self harm" in main search bar, filtered to show only results in the UK

Total hits = 91, of which 57 were duplicates

Results which met eligibility criteria for full-text inclusion:

#	Webpage Name	Link
1	Understanding and addressing the mental health needs of adolescents	http://www.euro.who.int/en/countries/belarus/news/news/2018/01/understanding-and-addressing-the-mental-health-needs-of-adolescents
2	For which strategies of suicide prevention is there evidence of effectiveness?	http://www.euro.who.int/en/data-and-evidence/evidence-informed-policy-making/publications/pre2009/for-which-strategies-of-suicide-prevention-is-there-evidence-of-effectiveness
3	Anna's story	http://www.euro.who.int/en/health-topics/noncommunicable-diseases/mental-health/data-and-resources/personal-stories/annas-story
4	Northern Ireland, United Kingdom: Caitriona Cassidy	http://www.euro.who.int/en/health-topics/Health-systems/pages/personal-stories/people-centred-health-systems-voices-of-patients-and-carers/northern-ireland,-united-kingdom-caitriona-cassidy
5	New WHO publication encourages investment in the health of adolescents in Europe – Adoelscent health in the European Union: can we do better?	http://www.euro.who.int/__data/assets/pdf_file/0005/407219/AA-HA-adaptation-V7_maket_10.07.19_e_book_2.pdf?ua=1
6	Wilma's story	http://www.euro.who.int/en/health-topics/noncommunicable-diseases/mental-health/data-and-resources/personal-stories/wilmas-story
7	Integrating the prevention, treatment and care of mental health conditions and other	http://www.euro.who.int/__data/assets/pdf_file/0004/397786/Mental-Health-Conditions-ENG.pdf?ua=1

	non-communicable diseases within health systems	
8	Mental health: facing the challenges, building solutions	http://www.euro.who.int/__data/assets/pdf_file/0008/96452/E87301.pdf

10. Public Health England

Link: https://www.gov.uk/government/organisations/public-health-england

Date searched: 24/9/19

Search strategy:

- Website browsed
- Individually searched "self harm", "self injury", "parasuicide", "self cutting", "self poisoning", "self injurious behaviour" and "deliberate self harm" in main search bar, filtered to only show 'guidance and regulation, 'research and statistics' and 'policy papers and consultations'

Total hits = 86, of which 4 were duplicates

Results which met eligibility criteria for full-text inclusion:

#	Webpage Name	Link
1	Intentional self-poisoning by young people	https://www.gov.uk/government/publications/intentional-self-poisoning-by-young-people
2	Self harm reduction strategy	https://www.gov.uk/government/publications/assessing-care-in-detention-self-harm-reduction-strategy
3	Suicide and self-harm prevention in prison	https://www.gov.uk/guidance/suicide-self-harm-prevention-in-prison
4	Mental health and self-harm in children and young people	https://www.gov.uk/government/publications/mental-health-and-self-harm-in-children-and-young-people
5	Self-harm by adult men in prison: a rapid evidence assessment	https://www.gov.uk/government/publications/self-harm-by-adult-men-in-prison-a-rapid-evidence-assessment

11. Public Health Scotland

Link: https://www.gov.scot/

Date searched: 24/9/19

Search strategy:

- Website browsed
- Individually searched "self harm", "self injury", "parasuicide", "self injurious behaviour", "self cutting", "self poisoning", and "deliberate self harm" in main search bar

Total hits = 79, of which 7 were duplicates

Results which met eligibility criteria for full-text inclusion:

#	Webpage Name	Link
1	Working with children and adults who may be at risk of self-harm: practical guidance	https://www.gov.scot/publications/working-children-adults-risk-self-harm-practice-guidance-information-sharing-protection-confidentiality/ https://www.webarchive.org.uk/wayback/archive/20180516085145/http://www.gov.scot/Publications/2011/03/17153551/2 https://www.webarchive.org.uk/wayback/archive/20180516085145/http://www.gov.scot/Publications/2011/03/17153551/2
2	Child and adolescent mental health	https://www.gov.scot/news/child-and-adolescent-mental-health/

12. Public Health Wales

Link: http://www.wales.nhs.uk/sitesplus/888/home

Date searched: 24/9/19

Search strategy:

- Website browsed
- Individually searched "self harm", "self injury", "parasuicide", "self injurious behaviour", "self cutting", "self poisoning" and "deliberate self harm" in main search bar, with search terms entered into the 'exact phrase' box, filtered to show only pages and documents

Total hits = 58, of which 10 were duplicates

Results which met eligibility criteria for full-text inclusion:

#	Webpage Name	Link
1	Talk to me: Mid Point Review	No URL available as downloaded as Microsoft Word document
2	Suicide and self harm	http://www.wales.nhs.uk/sitesplus/888/page/65108/

13. HSC Public Health Agency

Link: https://www.publichealth.hscni.net/

Date searched: 24/9/19

Search strategy:

- Website browsed
- Individually searched "self harm", "self injury", "parasuicide", "self injurious behaviour", "deliberate self harm", "self cutting" and "self poisoning" in main search bar

Total hits = 23, of which 0 were duplicates

Results which met eligibility criteria for full-text inclusion:

#	Webpage Name	Link
1	PHA published 3 year report into self-harm in Northern Ireland	https://www.publichealth.hscni.net/news/pha-publishes-three-year-report-self-harm-northern-ireland
2	Public Health Agency Self-Harm Symposium Conference Report	https://www.publichealth.hscni.net/publications/public-health-agency-self-harm-symposium-conference-report
3	Improving the lives of people who self harm	https://www.publichealth.hscni.net/publications/improving-lives-people-who-self-harm
4	What happens in the Emergency Department, a patient's guide	https://www.publichealth.hscni.net/publications/what-happens-emergency-department-patient%E2%80%99s-guide
5	Evaluation of Pilot One Stop Shop Programme	https://www.publichealth.hscni.net/publications/evaluation-pilot-one-stop-shop-programme
6	NI Registry of Self-Harm Annual Report 2012/2013	https://www.publichealth.hscni.net/publications/ni-registry-self-harm-annual-report-201213
7	Northern Ireland of Self-Harm Western Are Six Year Summary Report 2007-2012	https://www.publichealth.hscni.net/publications/northern-ireland-registry-self-harm-western-area-six-year-summary-report-2007%E2%80%932012
8	Northern Ireland Registry of Self-Harm Annual Report 2017-2018	https://www.publichealth.hscni.net/publications/northern-ireland-registry-self-harm-annual-report-2017-2018
9	Community Based Psychological Intervention and Support Services for People who Self-harm including	https://www.publichealth.hscni.net/contracts/tenders/community-based-psychological-intervention-and-support-services-people-who-self-ha

	provision of family/carer services	
10	PHA published six year report on self-harm in western area	https://www.publichealth.hscni.net/news/pha-publishes-six-year-report-self-harm-western-area
11	Services available for people who self-harm	https://www.publichealth.hscni.net/news/services-available-people-who-self-harm

14. Royal Society for Public Health

Link: https://www.rsph.org.uk/

Date searched: 20/9/19

Search strategy:

- Website browsed
- Individually searched "self harm", "self injury", "parasuicide", "self injurious behaviour", "deliberate self harm", "self cutting" and "self poisoning" in main search bar

Total hits = 147, of which 74 were duplicates

Results which met eligibility criteria for full-text inclusion:

#	Webpage Name	Link
1	Looking after your self-ie	https://www.rsph.org.uk/our-services/e-learning/courses/free-courses/looking-after-your-selfie.html
2	Guest blog: self-care for all	https://www.rsph.org.uk/about-us/news/guest-blog-self-care-for-all.html
3	Guest blog: self-care – everyone's default setting?	https://www.rsph.org.uk/about-us/news/guest-blog-self-care-everyone-s-default-setting.html

15. Centre for Mental Health

Link: https://www.centreformentalhealth.org.uk/

Date searched: 20/9/19

Search strategy:

- Website browsed
- Individually searched "self harm", "self-harm", "self injury", "parasuicide", "self cutting", "self poisoning", "deliberate self harm" and "self injurious behaviour" in main search bar

Total hits = 30, of which 0 were duplicates

Results which met eligibility criteria for full-text inclusion:

#	Webpage Name	Link
1	NHS and local authorities should invest in support for young people who self-harm, says Centre for Mental Health report	https://www.centreformentalhealth.org.uk/news/invest-young-people-self-harm
2	A space to talk	https://www.centreformentalhealth.org.uk/a-space-to-talk
3	The children's mental health survey and its implications	https://www.centreformentalhealth.org.uk/blog/childrens-mental-health-survey
4	Therapy dogs should be more widely available in prisons to improve wellbeing and self-harm, says Centre for Mental Health report	https://www.centreformentalhealth.org.uk/news/therapy-dogs-should-be-more-widely-available-prisons

5	Bringing mums' minds from darkness to light: Sadia's story	https://www.centreformentalhealth.org.uk/blog/centre-mental-health-blog/bringing-mums-minds-darkness-light-sadias-story
6	Investing in children's mental health	https://www.centreformentalhealth.org.uk/publications/investing-in-childrens-mental-health
7	Can social media help young people's emotional wellbeing?	https://www.centreformentalhealth.org.uk/blog/centre-mental-health-blog/social-media-young-peoples-wellbeing

16. British Association for Behavioural and Cognitive Psychotherapies

Link: https://www.babcp.com/Default.aspx
Date searched: 20/8/19
Search strategy:

- Website browsed
- No search bar identified

Total hits = 0

17. British Association for Counselling and Psychotherapy

Link: https://www.bacp.co.uk/
Date searched: 20/8/19
Search strategy:

- Website browsed
- Individually searched "self harm", "self injury", "parasuicide", "self cutting", "self poisoning", "deliberate self harm" and "self injurious behaviour" in main search bar, filtered to only show 'articles' and 'guidance', excluded member-only content

Total hits = 62, of which 3 were duplicates
Results which met eligibility criteria for full-text inclusion:

#	**Webpage Name**	**Link**
1	Busting the myths and misconceptions about self-harm	https://www.bacp.co.uk/news/news-from-bacp/2019/1-march-busting-the-myths-and-misconceptions-about-self-harm/
2	"Worrying" new figures show more than half of people who self-harm do not access support	https://www.bacp.co.uk/news/news-from-bacp/2019/5-june-worrying-new-figures-show-more-than-half-of-people-who-self-harm-do-not-access-support/
3	Bringing the 'self' into self-harm	https://www.bacp.co.uk/bacp-journals/university-and-college-counselling/november-2014/bringing-the-self-into-self-harm/
4	Sticky bubbles, stats and Snap	https://www.bacp.co.uk/bacp-journals/bacp-children-young-people-and-families-journal/september-2013/sticky-bubbles-stats-and-snap/
5	'Counselling has helped me find out who I am – and be comfortable in my own skin'	https://www.bacp.co.uk/news/news-from-bacp/2019/16-may-counselling-has-helped-me-find-out-who-i-am-and-be-comfortable-in-my-own-skin/
6	Suicide and self harm: psychological therapies, prevention and risk factors	https://www.bacp.co.uk/media/2128/bacp-suicide-self-harm-briefing-oct15.pdf
7	What therapy can help with	https://www.bacp.co.uk/about-therapy/what-therapy-can-help-with/

18. UK Council for Psychotherapy

Link: https://www.psychotherapy.org.uk/
Date searched: 20/8/19

Search strategy:

- Website browsed
- Individually searched "self harm", "self injury", "parasuicide", "self injurious behaviour", "deliberate self harm", "self cutting" and "self poisoning" in main search bar

Total hits = 13, of which 3 were duplicates

Results which met eligibility criteria for full-text inclusion:

#	Webpage Name	Link
1	One in six children report self-harming by age 14	https://www.psychotherapy.org.uk/ukcp-news/midweek-mindset/1-in-6-children-report-self-harming-by-age-14/
2	Millennial mind set: Bristol University students protest the 'growing mental health crisis'	https://www.psychotherapy.org.uk/ukcp-news/millennial-mindset/bristol-university-students-protest-the-growing-mental-health-crisis/
3	Official NHS data reveals rise in mental health issues among children	https://www.psychotherapy.org.uk/ukcp-news/millennial-mindset/official-nhs-data-reveals-rise-in-mental-health-issues-among-children/
4	Referrals to children and adolescent services up by 26%	https://www.psychotherapy.org.uk/ukcp-news/midweek-mindset/referrals-to-children-and-adolescent-services-up-by-26/

19. British Psychological Society

Link: https://www.bps.org.uk/

Date searched: 18/9/19

Search strategy:

- Website browsed
- Individually searched "self harm", "self injury", "parasuicide", "self injurious behaviour", "deliberate self harm", "self cutting" and "self poisoning" in main search bar

Total hits = 4,718

- Due to high volume of results and decreasing relevance, only the first 100 search results for each search term were screened

Modified hits = 141, of which 16 were duplicates

Results which met eligibility criteria for full-text inclusion:

#	Webpage Name	Link
1	BPS responds to media reports on self-harming by teenage girls and its links to social media use	https://www.bps.org.uk/news-and-policy/bps-responds-media-reports-self-harming-teenage-girls-and-its-links-social-media-use
2	BPS endorses new competence frameworks for the care of people at risk of self-harm and suicide	https://www.bps.org.uk/news-and-policy/bps-endorses-new-competence-frameworks-care-people-risk-self-harm-and-suicide
3	Family support in self-harming	https://www.bps.org.uk/news-and-policy/family-support-self-harming
4	A-Z psychology	https://www.bps.org.uk/public/a-z-of-psychology

20. Samaritans

Link: https://www.samaritans.org/

Date searched: 29/8/19

Search strategy:

- Website browsed

- Individually searched “self harm”, “self injury”, “parasuicide”, “self injurious behaviour”, “deliberate self harm”, “self cutting” and “self poisoning” in main search bar

Total hits = 96, of which 7 were duplicates

Results which met eligibility criteria for full-text inclusion:

#	Webpage Name	Link
1	Self-harm – Samaritans offer support	https://www.samaritans.org/branches/ballymena/samaritans-ballymena-news/self-harm-samaritans-offer-support/
2	DEAL: self-harm myths and facts	https://www.samaritans.org/how-we-can-help/schools/deal/deal-resources/dealing-feelings/self-harm-myths-and-facts/
3	Practical ways to help yourself cope	https://www.samaritans.org/how-we-can-help/support-and-information/if-youre-having-difficult-time/signs-you-may-be-struggling-cope/practical-ways-help-yourself-cope/

21. SANE

Link: http://www.sane.org.uk/home

Date searched: 29/8/19

Search strategy:

- Website browsed (1 hit)
- Individually searched “self harm”, “self injury”, “parasuicide”, “self injurious behaviour”, “deliberate self harm”, “self cutting” and “self poisoning” in main search bar

Total hits = 3, of which 0 were duplicates

Results which met eligibility criteria for full-text inclusion:

#	Webpage Name	Link
1	Self harm	http://www.sane.org.uk/uploads/Self_Harm-1.pdf
2	Self harm research	http://www.sane.org.uk/resources/self_harm/

22. CALM

Link: https://www.thecalmzone.net/

Date searched: 29/8/19

Search strategy:

- Website browsed (1 hit)
- Individually searched “self harm”, “self injury”, “parasuicide”, “self injurious behaviour”, “deliberate self harm”, “self cutting” and “self poisoning” in main search bar

Total hits = 22, of which 1 was a duplicate

Results which met eligibility criteria for full-text inclusion:

#	Webpage Name	Link
1	Self harm	https://www.thecalmzone.net/help/get-help/self-harm/
2	Self harm and short sleeves	https://www.thecalmzone.net/2013/08/selfharmshortsleeves/?highlight=%22self%20harm%22

23. Childline

Link: https://www.childline.org.uk/

Date searched: 29/8/19

Search strategy:

- Website browsed (4 hits)
- Individually searched “self harm”, “self injury”, “parasuicide”, “self injurious behaviour”, “deliberate self harm”, “self cutting” and “self poisoning” in main search bar, filtered to show ‘content listings’, ‘articles’ and ‘explore’

Total hits = 25, of which 4 were duplicates

Results which met eligibility criteria for full-text inclusion:

#	Webpage Name	Link
1	Self harm coping techniques	https://www.childline.org.uk/info-advice/your-feelings/self-harm/self-harm-coping-techniques/
2	Self harm	https://www.childline.org.uk/info-advice/your-feelings/self-harm/self-harm/
3	Advice about helping a friend	https://www.childline.org.uk/info-advice/friends-relationships-sex/friends/helping-friend/
4	Coping with stress	https://www.childline.org.uk/info-advice/your-feelings/anxiety-stress-panic/coping-with-stress/
5	Helena’s story	https://www.childline.org.uk/get-involved/real-life-stories/self-harm-helenas-story/
6	Trauma: Adam’s story	https://www.childline.org.uk/get-involved/real-life-stories/trauma-adams-story/
7	Depression and feeling sad	https://www.childline.org.uk/info-advice/your-feelings/feelings-emotions/depression-feeling-sad/

24. SelfHarmUK

Link: https://www.selfharm.co.uk/

Date searched: 2/9/19

Search strategy:

- Website browsed (20 hits)
- Individually searched “self harm”, “self injury”, “parasuicide”, “self injurious behaviour”, “deliberate self harm”, “self cutting” and “self poisoning” in main search bar

Total hits = 133, of which 23 were duplicates

Results which met eligibility criteria for full-text inclusion:

#	Webpage Name	Link
1	What is self-harm?	https://www.selfharm.co.uk/get-information/the-facts/what-is-self-harm
2	Who self-harms?	https://www.selfharm.co.uk/get-information/the-facts/who-self-harms
3	Self harm statistics	https://www.selfharm.co.uk/get-information/the-facts/self-harm-statistics
4	Recovering from self-harm	https://www.selfharm.co.uk/get-information/the-facts/recovering-from-self-harm
5	Dealing with scars	https://www.selfharm.co.uk/get-information/the-facts/dealing-with-scars
6	Disclosure: sharing stories of self-harm	https://www.selfharm.co.uk/get-information/the-facts/disclosure-sharing-stories-of-self-harm
7	Autism and self-harm	https://www.selfharm.co.uk/get-information/the-facts/autism-and-self-harm
8	Eating disorders and self-harm	https://www.selfharm.co.uk/get-information/the-facts/eating-disorders-and-self-harm
9	Boys and self-harm	https://www.selfharm.co.uk/get-information/the-facts/boys-and-self-harm
10	Self-harm in LGBTQI young people	https://www.selfharm.co.uk/get-information/the-facts/self-harm-in-lgbtqi-young-people

11	SEN and self-harm	https://www.selfharm.co.uk/get-information/the-facts/sen-and-self-harm
12	They must enjoy it	https://www.selfharm.co.uk/get-information/the-myths/they-must-enjoy-it
13	Self-harmers must be mentally ill	https://www.selfharm.co.uk/get-information/the-myths/self-harmers-must-be-mentally-ill
14	Self-harm and suicide	https://www.selfharm.co.uk/get-information/the-myths/self-harm-and-suicide
15	Attention, please	https://www.selfharm.co.uk/get-information/the-myths/attention-please
16	It's all the internet's fault	https://www.selfharm.co.uk/get-information/the-myths/its-all-the-internets-fault
17	Knowing your body	https://www.selfharm.co.uk/get-information/staying-safe/knowing-your-body
18	Harm minimisation	https://www.selfharm.co.uk/get-information/staying-safe/harm-minimisation
19	Poisoning	https://www.selfharm.co.uk/get-information/staying-safe/poisoning
20	Looking after you	https://www.selfharm.co.uk/get-information/staying-safe/looking-after-you
21	Self-harm triggers	https://www.selfharm.co.uk/blog/self-harm-triggers
22	Living with self-harm	https://www.selfharm.co.uk/blog/living-with-self-harm
23	Self-harm is real	https://www.selfharm.co.uk/blog/self-harm-is-real
24	Kelly's battle with self-harm	https://www.selfharm.co.uk/blog/kellys-battle-with-self-harm
25	How to get control over your self-harm	https://www.selfharm.co.uk/blog/how-to-get-control-over-your-self-harm
26	Will alternatives work?	https://www.selfharm.co.uk/blog/will-alternatives-work
27	Health Habits	https://www.selfharm.co.uk/blog/healthy-habits
28	Recovery – the Long Road and what I wish someone would have told me	https://www.selfharm.co.uk/blog/recovery-the-long-road
29	Alternatives and distractions	https://www.selfharm.co.uk/blog/alternatives-and-distractions
30	It's better to talk	https://www.selfharm.co.uk/blog/its-better-to-talk

25. LifeSIGNS

Link: http://www.lifesigns.org.uk/

Date searched: 19/9/19

Search strategy:

- Website browsed (13 hits)
- Individually searched "self harm", "self injury", "parasuicide", "self injurious behaviour", "deliberate self harm", "self cutting" and "self poisoning" in main search bar

Total hits = 963

- Due to high volume of results and decreasing relevance, only the first 100 search results for each search term were screened

Modified hits = 222, of which 81 were duplicates

Results which met eligibility criteria for full-text inclusion:

#	**Webpage Name**	**Link**
1	Helping you	http://www.lifesigns.org.uk/help/
2	Read this first	http://www.lifesigns.org.uk/read-this-first/
3	HALT	http://www.lifesigns.org.uk/halt/
4	Alternatives	http://www.lifesigns.org.uk/alternatives/
5	15 minute rule	http://www.lifesigns.org.uk/15-minutes-rule/

6	Surfing the urge	http://www.lifesigns.org.uk/surfing-the-urge/
7	Everything but rule	http://www.lifesigns.org.uk/everything-but-rule/
8	Distraction box	http://www.lifesigns.org.uk/distraction-box/
9	Masturbation	http://www.lifesigns.org.uk/masturbation/
10	Choice	http://www.lifesigns.org.uk/choice/
11	Male SI	http://www.lifesigns.org.uk/male-si/
12	Invalidation and criticism	http://www.lifesigns.org.uk/addressing-invalidation-criticism-feedback-and-self-criticism-with-cbt/
13	Getting help	http://www.lifesigns.org.uk/getting-help/
14	Why is it so hard to stop?	http://www.lifesigns.org.uk/why-is-it-so-hard-to-stop/
15	How to react when your friend says they self-injure	http://www.lifesigns.org.uk/how-to-react-when-your-friend-says-they-self-injure/
16	New year new you? Goals, not resolutions, will help you move away from self-injury	http://www.lifesigns.org.uk/2018/01/new-year-new-you-goals-not-resolutions-will-help-you-move-away-from-self-injury/
17	Self poisoning	http://www.lifesigns.org.uk/2017/05/self-poisoning/
18	Drugs and self-recovery – Ben's ongoing recovery	http://www.lifesigns.org.uk/2016/07/drugs-and-self-injury-bens-on-going-recovery/
19	Self-injury addiction	http://www.lifesigns.org.uk/self-injury-addiction/
20	No harm 'contracts'	http://www.lifesigns.org.uk/2013/04/no-harm-contracts/
21	Self-injury: self expression inside out – hard to stop	http://www.lifesigns.org.uk/2010/02/self-injury-self-expression-inside-out-hard-to-stop/
22	Adult self-injury	http://www.lifesigns.org.uk/adult-self-injury/
23	Self-injury: the choice to cope and survive	http://www.lifesigns.org.uk/2013/10/self-injury-the-choice-to-cope-and-survive/
24	Terrible times, self-injury and recovery – by Kaveeta	http://www.lifesigns.org.uk/2013/02/terrible-times-self-injury-and-recovery-by-kaveeta/
25	Andy's self-injury research results summary	http://www.lifesigns.org.uk/2010/06/andys-self-injury-research-results-summary/
26	FAQs about self-injury	http://www.lifesigns.org.uk/2010/05/faqs-about-self-injury/
27	Self-Injury : self expression inside out – distraction and other alternatives	http://www.lifesigns.org.uk/2010/03/self-injury-self-expression-inside-out-distraction-and-other-alternatives/
28	Helping you with self-harm	http://www.lifesigns.org.uk/2014/11/helping-you-with-self-harm/
29	"You kept me from seriously harming myself tonight"	http://www.lifesigns.org.uk/2011/12/you-kept-me-from-seriously-harming-myself-tonight/
30	NICE consults on draft recommendations for the longer-term management of self-harm	http://www.lifesigns.org.uk/2011/04/nice-consults-on-draft-recommendations-for-thelonger-term-management-of-self-harm/
31	Dr Neil joins LifeSIGNS to offer psychiatric guidance to people who self-injure	http://www.lifesigns.org.uk/2008/02/dr-neil-joins-lifesigns-to-offer-psychiatric-guidance-to-people-who-self-injure/

26. Harmless

Link: http://www.harmless.org.uk/

Date searched: 2/9/19

Search strategy:

- Website browsed (3 hits)
- No search bar identified

Total hits = 3

Results which met eligibility criteria for full-text inclusion:

#	Webpage Name	Link

1	People who self-harm	http://www.harmless.org.uk/whoWeSupport/peopleWhoSelfHarm
2	Family and friends	http://www.harmless.org.uk/downloads/factSheet2_AdviceForFriendsAndFamily.pdf
3	Young people	http://www.harmless.org.uk/downloads/factSheet1_AdviceForYoungPeople.pdf

27. Recover Your Life

Link: http://www.recoveryourlife.com/
Date searched: 3/9/19
Search strategy:

- Website browsed (8 hits)
- No search bar identified

Total hits = 8
Results which met eligibility criteria for full-text inclusion:

#	Webpage Name	Link
1	The underlying causes	http://www.recoveryourlife.com/index.php?categoryid=22
2	You are not alone	http://www.recoveryourlife.com/index.php?categoryid=23
3	Preparing to stop	http://www.recoveryourlife.com/index.php?categoryid=64
4	Distraction advice	http://www.recoveryourlife.com//index.php?categoryid=61
5	Seeking professional help	http://www.recoveryourlife.com/index.php?categoryid=65
6	Sticking to stopping	http://www.recoveryourlife.com/index.php?categoryid=66

28. Inspire Wellbeing

Link: https://www.inspirewellbeing.org/
Date searched: 3/9/19
Search strategy:

- Website browsed
- No search bar identified

Total hits = 0

29. Papyrus

Link: https://papyrus-uk.org/
Date searched: 29/8/19
Search strategies:

- Website browsed
- Individually searched "self harm", "self injury", "parasuicide", "self injurious behaviour", "deliberate self harm", "self cutting" and "self poisoning" in main search bar

Total hits = 17, of which 2 were duplicates
Results which met eligibility criteria for full-text inclusion:

#	Webpage Name	Link
1	Self-injury Awareness Day	https://papyrus-uk.org/self-injury-awareness-day/
2	Wellbeing Apps	https://papyrus-uk.org/wellbeing-apps/

30. BEAT

Link: https://www.beateatingdisorders.org.uk/

Date searched: 4/9/19

Search strategies:

- Website browsed
- Individually searched "self -harm", "self-injury", "parasuicide", "self-injurious behaviour", "deliberate self-harm", "self-cutting" and "self-poisoning" in main search bar (no results found without hyphons)

Total hits = 196, of which 45 were duplicates

Results which met eligibility criteria for full-text inclusion:

#	Webpage Name	Link
1	Keep pushing forward and everything will fall into place	https://www.beateatingdisorders.org.uk/your-stories/keep-pushing-forward-everything-fall-into-place
2	Overturning bad decisions and understanding appropriate ones	https://www.beateatingdisorders.org.uk/support-services/overturning-bad-decisions
3	My journey to recovery and beyond	https://www.beateatingdisorders.org.uk/your-stories/my-journey-to-recovery-and-beyond
4	Advice for recovery from someone who has been there	https://www.beateatingdisorders.org.uk/your-stories/advice-recovery-been-there
5	Aim for progress, not perfection	https://www.beateatingdisorders.org.uk/your-stories/recovery/progress-not-perfection
6	Recovery: it's never too late	https://www.beateatingdisorders.org.uk/your-stories/recovery/recovery-never-too-late
7	To recover, I needed to be okay with being me	https://www.beateatingdisorders.org.uk/your-stories/to-recover-needed-okay-with-me
8	Honour your body for all it does for you	https://www.beateatingdisorders.org.uk/your-stories/honour-your-body-for-all-it-does-for-you
9	Why the journey to recovery is just as important as the outcome	https://www.beateatingdisorders.org.uk/your-stories/why-journey-recovery-important-outcome
10	When does recovery become recovered?	https://www.beateatingdisorders.org.uk/your-stories/when-does-recovery-become-recovered
11	From eating disorders and self-harm to health and happiness	https://www.beateatingdisorders.org.uk/your-stories/from-eating-disorders-self-harm-health-happiness
12	I decided to stop with the denial and start my path to recovery	https://www.beateatingdisorders.org.uk/your-stories/recovery/stop-denial-start-recovery
13	From surviving to thriving: 5 years on	https://www.beateatingdisorders.org.uk/your-stories/recovery/surviving-thriving-five-years-on
14	Real, raw, recovery… It's all about the little things	https://www.beateatingdisorders.org.uk/your-stories/recovery/real-raw-recovery-its-about-little-things
15	My fight for recovery	https://www.beateatingdisorders.org.uk/your-stories/anorexia/my-fight-for-recovery
16	Recovery information	https://www.beateatingdisorders.org.uk/recovery-information

31. Rethink Mental Illness

Link: https://www.rethink.org

Date searched: 4/9/19

Search strategy:

- Website browsed (1 hit)
- Individually searched "self harm", "self injury", "parasuicide", "self injurious behaviour", "deliberate self harm", "self cutting" and "self poisoning" in main search bar

Total hits = 305, of which 252 were duplicates

Results which met eligibility criteria for full-text inclusion:

#	Webpage Name	Link
1	Prisoners and self harm	https://www.rethink.org/advice-and-information/rights-restrictions/police-courts-and-prison/prisoners-and-self-harm/
2	Therapy dogs should be more widely available	https://www.rethink.org/news-and-stories/news/2018/dec/therapy-dogs-should-be-more-widely-available-in-prisons-to-improve-wellbeing-and-self-harm-says-new-report/
3	Largest survey of its kind…	https://www.rethink.org/news-and-stories/news/2019/mar/largest-survey-of-its-kind-reveals-extent-of-university-students-struggles-with-thoughts-of-self-harm-loneliness-and-anxiety/
4	Today marks one year since I last self-harmed	https://www.rethink.org/news-and-stories/blogs/2018/dec/today-marks-one-year-since-i-last-self-harmed/

32. Mind

Link: https://www.mind.org.uk/

Date searched: 29/8/19

Search strategy:

- Website browsed
- Individually searched "self harm", "self injury", "parasuicide", "self injurious behaviour", "deliberate self harm", "self cutting" and "self poisoning" in main search bar

Total hits = 110, of which 4 were duplicates

Results which met eligibility criteria for full-text inclusion:

#	Webpage Name	Link
1	Self-harm	https://www.mind.org.uk/information-support/types-of-mental-health-problems/self-harm/#.XWeLDC5KiUk
2	Helping yourself now	https://www.mind.org.uk/information-support/types-of-mental-health-problems/self-harm/helping-yourself-now/#.XWeLXy5KiUk (29/8/19)
3	Why people self harm	https://www.mind.org.uk/information-support/types-of-mental-health-problems/self-harm/why-people-self-harm/#.XWeLfy5KiUk
4	For friends and family	https://www.mind.org.uk/information-support/types-of-mental-health-problems/self-harm/for-friends-and-family/#.XWeLny5KiUk
5	Treatment and support	https://www.mind.org.uk/information-support/types-of-mental-health-problems/self-harm/treatment-and-support/#.XWeLxC5KiUk
6	Helping yourself long-term	https://www.mind.org.uk/information-support/types-of-mental-health-problems/self-harm/helping-yourself-long-term/#.XWeMhS5KiUk
7	Self-care for BPD	https://www.mind.org.uk/information-support/types-of-mental-health-problems/borderline-personality-disorder-bpd/self-care-for-bpd/#.XWeMqS5KiUk
8	Self-care	https://www.mind.org.uk/information-support/types-of-mental-health-problems/personality-disorders/self-care/#.XWeNky5KiUk
9	Dialectical behaviour therapy	https://www.mind.org.uk/information-support/drugs-and-treatments/dialectical-behaviour-therapy-dbt/#.XWeNvi5KiUk
10	Difficult feelings & behaviours	https://www.mind.org.uk/information-support/types-of-mental-health-problems/mental-health-problems-introduction/difficult-feelings-behaviours/#.XWeOEi5KiUk
11	Treatment and support (BPD)	https://www.mind.org.uk/information-support/types-of-mental-health-problems/borderline-personality-disorder-bpd/treatment-and-support/#.XWePGy5KiUk
12	Treatments (depression)	https://www.mind.org.uk/information-support/types-of-mental-health-problems/depression/treatments/#.XWePfS5KiUk

13	Resilience	https://www.mind.org.uk/information-support/police/mental-wellbeing-police/resilience/#.XWeP7C5KiUk

33. Battle Scars

Link: https://www.battle-scars-self-harm.org.uk/

Date searched: 21/8/19

Search strategy:

- Website browsed
- Individually searched "self harm", "self injury", "parasuicide", "self injurious behaviour", "deliberate self harm", "self cutting" and "self poisoning" in main search bar

Total hits = 28, of which 0 were duplicates

Results which met eligibility criteria for full-text inclusion:

#	Webpage Name	Link
1	What is self-harm all about?	https://www.battle-scars-self-harm.org.uk/what-is-self-harm-all-about.html

34. Mental Health Matters

Link: https://www.mhm.org.uk/

Date searched: 4/9/19

Search strategy:

- Website browsed
- Individually searched "self harm", "self injury", "parasuicide", "self injurious behaviour", "deliberate self harm", "self cutting" and "self poisoning" in main search bar

Total hits = 389, of which 314 were duplicates

Results which met eligibility criteria for full-text inclusion:

#	Webpage Name	Link
1	What is CBT?	https://www.mhm.org.uk/what-is-cognitive-behavioural-therapy-cbt
2	Self-injury awareness day	https://www.mhm.org.uk/News/self-injury-awareness-day-1st-march-2019
3	Boosting self-esteem	https://www.mhm.org.uk/Blog/boosting-self-esteem
4	Spotting the signs	https://www.mhm.org.uk/Blog/spotting-the-signs
5	Road to recovery	https://www.mhm.org.uk/Blog/road-to-recovery

35. Youth Access

Link: https://www.youthaccess.org.uk/

Date searched: 4/9/19

Search strategy:

- Website browsed
- Individually searched "self harm", "self injury", "parasuicide", "self injurious behaviour", "deliberate self harm", "self cutting" and "self poisoning" in main search bar

Total hits = 20, of which 6 were duplicates

Results which met eligibility criteria for full-text inclusion:

#	Webpage Name	Link
1	Children and young people's views on counselling: improving the tools to gather outcomes	https://www.youthaccess.org.uk/downloads/childrenandyoungpeoplesviewoncounselling-march20141.pdf

2	Guide to induction for newly appointed counsellors in youth information, advice, counselling and support services	https://www.youthaccess.org.uk/downloads/httpwww.youthaccess.org.ukresourcesgoodpracticeloader.pdf

36. Mood Swings

Link: http://www.moodswings.org.uk/

Date searched: 5/9/19

Search strategy:

- Website browsed
- Individually searched "self harm", "self injury", "parasuicide", "self injurious behaviour", "deliberate self harm", "self cutting" and "self poisoning" in main search bar

Total hits = 0

37. No Panic

Link: https://www.nopanic.org.uk/

Date searched: 5/9/19

Search strategy:

- Website browsed (2 hits)
- No search bar identified

Total hits = 2

Results which met eligibility criteria for full-text inclusion:

#	Webpage Name	Link
1	Self-harm and suicidal thoughts	https://www.nopanic.org.uk/self-harm-suicidal-thoughts/
2	Recovery and self-help	https://www.nopanic.org.uk/recovery-self-help/

38. Life Centre

Link: https://lifecentre.uk.com/

Date searched: 5/9/19

Search strategy:

- Website browsed
- No search bar identified

Total hits = 0

39. Survivors UK

Link: https://www.survivorsuk.org/

Date searched: 5/9/19

Search strategy:

- Website browsed
- Individually searched "self harm", "self injury", "parasuicide", "self injurious behaviour", "deliberate self harm", "self cutting" and "self poisoning" in main search bar

Total hits = 8, of which 0 were duplicates

Results which met eligibility criteria for full-text inclusion:

#	Webpage Name	Link
1	Self-help guidance	https://www.survivorsuk.org/resources/self-help-guidance/ http://www.selfhelpguides.ntw.nhs.uk/survivorsmcr/leaflets/selfhelp/Self%20Harm.pdf

40. Self Injury Support Network

Link: https://www.selfinjurysupport.org.uk/

Date searched: 5/9/19

Search strategy:

- Website browsed
- Individually searched "self harm", "self injury", "parasuicide", "self injurious behaviour", "deliberate self harm", "self cutting" and "self poisoning" in main search bar

Total hits = 989, of which 822 were duplicates

Results which met eligibility criteria for full-text inclusion:

#	Webpage Name	Link
1	What is self-injury?	https://www.selfinjurysupport.org.uk/what-is-self-injury
2	Self care and self harm spectrum	https://www.selfinjurysupport.org.uk/self-care-and-self-harm-spectrum
3	Launch of self-harm prevention app distract	https://www.selfinjurysupport.org.uk/News/launch-of-self-harm-prevention-app-distract
4	Why do I self-injure?	https://www.selfinjurysupport.org.uk/why-do-i-self-injure
5	Why does it happen?	https://www.selfinjurysupport.org.uk/why-does-it-happen
6	What if I want to stop?	https://www.selfinjurysupport.org.uk/i-want-to-stop
7	Self-harm: a self help guide	https://www.selfinjurysupport.org.uk/FAQs/a-self-help-guide
8	Self-harm and sexual violence	https://www.selfinjurysupport.org.uk/FAQs/self-harm-and-sexual-violence
9	Self-harm information sheet	https://www.selfinjurysupport.org.uk/FAQs/self-harm-information-sheet
10	Reducing self-harm worksheet	https://www.selfinjurysupport.org.uk/FAQs/reducing-self-harm-worksheet
11	Working through self-harm	https://www.selfinjurysupport.org.uk/FAQs/working-through-self-harm
12	Self-harm in children and young people	https://www.selfinjurysupport.org.uk/FAQs/self-harm-in-children-and-young-people
13	Distraction and distancing from self-harm	https://www.selfinjurysupport.org.uk/FAQs/distraction-and-distancing
14	Self-harm in primary age children	https://www.selfinjurysupport.org.uk/FAQs/self-harm-in-primary-age-children
15	Self-harm: treating people differently and intervening early	https://www.selfinjurysupport.org.uk/FAQs/self-harm-treating-people-differently-and-intervening-early
16	Coping with self-harm: a guide for parents and carers	https://www.selfinjurysupport.org.uk/FAQs/coping-with-self-harm-a-guide-for-parents-and-carers
17	Self-harm support action plan accompanying notes	https://www.selfinjurysupport.org.uk/FAQs/self-harm-support-action-plan-accompanying-notes
18	What if I want to stop self-harming?	https://www.selfinjurysupport.org.uk/FAQs/what-if-i-want-to-stop-self-harming
19	Self-harm report card for seeking treatment	https://www.selfinjurysupport.org.uk/FAQs/self-harm-report-card
20	Self harm in children and young people handbook	https://www.selfinjurysupport.org.uk/FAQs/self-harm-in-children-and-young-people-handbook

21	Medical skin camouflage: a recovery intention for female prisoners who self-harm?	https://www.selfinjurysupport.org.uk/FAQs/medical-skin-camouflage-a-recovery-intervention-for-female-prisoners-who-self-harm
22	Managing self-harm in young people – Royal College of Psychiatrists	https://www.selfinjurysupport.org.uk/FAQs/managing-self-harm-in-young-people
23	Young people who self-harm: a guide for school staff	https://www.selfinjurysupport.org.uk/FAQs/young-people-who-self-harm-a-guide-for-school-staff
24	Supporting children and young people who self-harm: guidelines for school staff	https://www.selfinjurysupport.org.uk/FAQs/supporting-children-and-young-people-who-self-harm-guidelines-for-school-staff
25	NHS guidelines and resources for schools to help support children and young people who self-harm	https://www.selfinjurysupport.org.uk/FAQs/nhs-guidelines-and-resources-for-schools-to-help-support-children-and-young-people-who-self-harm
26	Guidance to providing effective support for self-harm amongst University students: a student's perspective	https://www.selfinjurysupport.org.uk/FAQs/guidance-to-providing-effective-support-for-self-harm-amongst-university-students-a-students-perspective
27	Self-harm factsheet for teachers and lecturers	https://www.selfinjurysupport.org.uk/FAQs/factsheet-for-teachers-and-lecturers
28	Self-harm minimisation in perspective: teaching and learning guidelines	https://www.selfinjurysupport.org.uk/FAQs/self-harm-minimisation-in-perspective-teaching-and-learning-guidelines
29	Self-harm – what, who, why and how to help	https://www.selfinjurysupport.org.uk/FAQs/self-harm-what-who-why-and-how-to-help
30	Working with young people who self-harm	https://www.selfinjurysupport.org.uk/FAQs/working-with-people-who-self-harm
31	Creative self-help ideas and strategies	https://www.selfinjurysupport.org.uk/creative-self-help
32	Self injury: what is it?	https://www.selfinjurysupport.org.uk/FAQs/self-injury-what-is-it
33	Understanding your self-injury	https://www.selfinjurysupport.org.uk/understanding-self-injury
34	Factsheet on self-injury for parents and guardians	https://www.selfinjurysupport.org.uk/FAQs/factsheet-on-self-injury-for-parents-and-guardians
35	Giving a voice to young people on self-injury	https://www.selfinjurysupport.org.uk/FAQs/giving-a-voice-to-young-people-on-self-injury
36	Self-injury and domestic violence information sheet	https://www.selfinjurysupport.org.uk/FAQs/self-injury-and-domestic-violence-information-sheet
37	Self-help for self-injury	https://www.selfinjurysupport.org.uk/FAQs/self-help-for-self-injury
38	Self-harm support action plan	https://www.selfinjurysupport.org.uk/FAQs/self-harm-support-action-plan-and-accompanying-notes
39	Exploring self-injury	https://www.selfinjurysupport.org.uk/FAQs/exploring-self-injury
40	Self-injury and autism	https://www.selfinjurysupport.org.uk/FAQs/self-injury-and-autism
41	Using mindfulness to distract from self-harm	https://www.selfinjurysupport.org.uk/FAQs/mindfulness-booklet
42	Factsheet on self-injury for health professionals	https://www.selfinjurysupport.org.uk/FAQs/factsheet-on-self-injury-for-health-professionals
43	Essential guide to self-injury and autism	https://www.selfinjurysupport.org.uk/FAQs/essential-guide-to-self-injurious-behaviour-and-autism
44	The relationship between self-injury and social media	https://www.selfinjurysupport.org.uk/FAQs/the-relationship-between-self-injury-and-social-media

45	Caring for someone who has self-harmed or had suicidal thoughts: a family guide	https://www.selfinjurysupport.org.uk/FAQs/caring-for-someone-who-has-self-harmed-or-had-suicidal-thoughts-a-family-guide
46	Predictors of self-injury cessation and subsequent psychological growth	https://www.selfinjurysupport.org.uk/FAQs/predictors-of-self-injury-cessation-and-subsequent-psychological-growth
47	Self-help resources for people with learning disabilities	https://www.selfinjurysupport.org.uk/self-help-resources-for-people-with-learning-disabilities
48	Distractions and alternatives	https://www.selfinjurysupport.org.uk/distractions-and-alternatives
49	Distractions that can help	https://www.selfinjurysupport.org.uk/FAQs/distractions-that-can-help
50	Information for parents: what you need to know about self-injury	https://www.selfinjurysupport.org.uk/FAQs/information-for-parents
51	The 'hurt yourself less' workbook	https://www.selfinjurysupport.org.uk/FAQs/the-hurt-yourself-less-workbook
52	Getting help	https://www.selfinjurysupport.org.uk/getting-help
53	Self-care booklet	https://www.selfinjurysupport.org.uk/FAQs/self-care-booklet
54	Distraction techniques and alternative coping strategies	https://www.selfinjurysupport.org.uk/FAQs/distraction-techniques-and-alternative-coping-strategies
55	Managing thoughts and feelings	https://www.selfinjurysupport.org.uk/thoughts-and-feelings

41. The Mix

Link: https://www.themix.org.uk/

Date searched: 13/9/19

Search strategy:

- Website browsed (23 hits)
- Individually searched "self harm", "self injury", "parasuicide", "self injurious behaviour", "deliberate self harm", "self cutting" and "self poisoning" in main search bar

Total hits = 47, of which 9 were duplicates

Results which met eligibility criteria for full-text inclusion:

#	Webpage Name	Link
1	Not all self-harm looks like self-harm	https://www.themix.org.uk/mental-health/self-harm/not-all-self-harm-looks-like-self-harm-31094.html
2	We need to talk about online self-harm content	https://www.themix.org.uk/mental-health/self-harm/we-need-to-talk-about-online-self-harm-content-30991.html
3	Self harm is an addictive way to cope with mental pain	https://www.themix.org.uk/mental-health/self-harm/self-harm-is-an-addictive-way-to-cope-with-mental-pain-nonormal-29249.html
4	Expert chat: more about self-harm	https://www.themix.org.uk/mental-health/self-harm/expert-chat-more-about-self-harm-16446.html (
5	Expert chat: self-harm	https://www.themix.org.uk/mental-health/self-harm/expert-chat-self-harm-13811.html
6	Child abuse made me self-harm	https://www.themix.org.uk/sex-and-relationships/family-life/childhood-abuse-made-me-self-harm-11644.html
7	Expert chat: how I cope with self-harm?	https://www.themix.org.uk/mental-health/expert-chat-how-do-i-cope-with-self-harm-9257.html
8	Having a self-harm relapse	https://www.themix.org.uk/mental-health/self-harm/having-a-self-harm-relapse-5684.html
9	Should I take antidepressants?	https://www.themix.org.uk/mental-health/anxiety-ocd-and-phobias/should-i-take-antidepressants-6119.html

10	Coping with their self-harm	https://www.themix.org.uk/mental-health/self-harm/coping-with-self-harm-5692.html
11	I keep on overdosing, what can I do?	https://www.themix.org.uk/mental-health/self-harm/i-keep-on-overdosing-what-can-i-do-6041.html
12	How to talk about your mental health	https://www.themix.org.uk/mental-health/looking-after-yourself/how-to-talk-about-your-mental-health-5622.html
13	Dealing with urges to self-harm	https://www.themix.org.uk/mental-health/self-harm/dealing-with-urges-to-self-harm-5698.html
14	Steps to self-harm recovery	https://www.themix.org.uk/mental-health/self-harm/steps-to-self-harm-recovery-5697.html
15	Self-harm coping tips and distractions	https://www.themix.org.uk/mental-health/self-harm/self-harm-coping-tips-and-distractions-5696.html
16	Self-harm myths	https://www.themix.org.uk/mental-health/self-harm/self-harm-myths-5695.html
17	Self-poisoning and overdosing	https://www.themix.org.uk/mental-health/depression-mental-health/self-poisoning-and-overdosing-5689.html
18	Their self-harm is too much for me	https://www.themix.org.uk/mental-health/self-harm/their-self-harm-is-too-much-for-me-5693.html
19	When your boyfriend/girlfriend self-harms	https://www.themix.org.uk/mental-health/self-harm/when-your-boyfriend-girlfriend-self-harms-5691.html
20	Telling someone you self-harm	https://www.themix.org.uk/mental-health/self-harm/telling-someone-you-self-harm-5682.html
21	Self-harm and your relationships	https://www.themix.org.uk/mental-health/self-harm/self-harm-and-your-relationships-5681.html
22	What is self-harm?	https://www.themix.org.uk/mental-health/self-harm/what-is-self-harm-5679.html
23	I want to kill myself	https://www.themix.org.uk/mental-health/depression-mental-health/i-want-to-kill-myself-5651.html
24	Mental health is valid, important and deserves attention	https://www.themix.org.uk/mental-health/body-image-and-self-esteem/sometimes-its-hard-to-feel-human-but-you-deserve-help-29099.html
25	Young men are hidden self-harmers	https://www.themix.org.uk/news-and-research/news/young-men-are-hidden-self-harmers
26	Family therapy	https://www.themix.org.uk/sex-and-relationships/family-life/family-therapy-7961.html
27	Self-harm blog week: breaking the silence	https://www.themix.org.uk/news-and-research/blogs/self-harm-blog-week-breaking-the-silence
28	Local help, easily found	https://www.themix.org.uk/news-and-research/news/local-help-easily-found

42. Young Minds

Link: https://youngminds.org.uk/

Date searched: 17/9/19

Search strategy:

- Website browsed (1 hit)
- Individually searched "self harm", "self injury", "parasuicide", "self cutting" and "self poisoning" in main search bar

Total hits = 529, of which 319 were duplicates

Results which met eligibility criteria for full-text inclusion:

#	Webpage Name	Link
1	Self-harm	https://youngminds.org.uk/find-help/feelings-and-symptoms/self-harm/
2	Young men and self-harm	https://youngminds.org.uk/blog/young-men-and-self-harm/

3	My Story of Self-Harm Recovery	https://youngminds.org.uk/blog/my-story-of-self-harm-recovery/
4	Responding to self-harm video	https://youngminds.org.uk/resources/school-resources/responding-to-self-harm-video/
5	Self-harm: having the first conversation	https://youngminds.org.uk/resources/school-resources/self-harm-having-the-first-conversation/
6	No harm done: talking to young people about self-harm	https://youngminds.org.uk/blog/no-harm-done-talking-to-young-people-about-self-harm/
7	Self harm	https://youngminds.org.uk/ucommerce/self-harm/c-23/p-177/
8	Self harm: what young people say	https://youngminds.org.uk/resources/school-resources/self-harm-what-young-people-say/
9	Responding to self-harm guide	https://youngminds.org.uk/resources/school-resources/responding-to-self-harm-guide/
10	Supporting your child who is self-harming	https://youngminds.org.uk/find-help/for-parents/parents-guide-to-support-a-z/parents-guide-to-support-self-harm/
11	5 things you can do if someone tells you they are self-harming	https://youngminds.org.uk/blog/five-things-you-can-do-if-someone-tells-you-they-are-self-harming/

43. MindEd

Link: https://www.minded.org.uk/
Date searched: 21/8/19
Search strategy:

- Website browsed
- Individually searched "self harm", "self injury", "parasuicide", "self injurious behaviour", "deliberate self harm", "self cutting" and "self poisoning" in main search bar

Total hits = 1
No results met eligibility criteria for full-text screening

44. Child Protection Company

Link: https://www.childprotectioncompany.com/CPC/index.html
Date searched: 4/9/19
Search strategy:

- Website browsed
- Individually searched "self harm", "self injury", "parasuicide", "self injurious behaviour", "deliberate self harm", "self cutting" and "self poisoning" in main search bar

Total hits = 6, of which 0 were duplicates
Results which met eligibility criteria for full-text inclusion:

#	Webpage Name	Link
1	Understanding self-harm in young people	https://www.childprotectioncompany.com/CPC/news/general/understanding-self-harm-in-young-people/

45. The Silver Line

Link: https://www.thesilverline.org.uk/
Date searched: 4/9/19
Search strategy:

- Website browsed
- No search bar identified

Total hits = 0

46. Adullam Ministries

Link: http://www.adullam-ministries.org.uk/site/

Date searched: 3/9/19

Search strategy:

- Website browsed (7)
- Individually searched “self harm”, “self injury”, “parasuicide”, “self injurious behaviour”, “deliberate self harm”, “self cutting” and “self poisoning” in main search bar

Total hits = 13, of which 3 were duplicates

Results which met eligibility criteria for full-text inclusion:

#	Webpage Name	Link
1	What is self harm?	http://www.adullam-ministries.org.uk/site/self-harm/what-is-self-harm/
2	Who self harms?	http://www.adullam-ministries.org.uk/site/self-harm/self-harms/
3	Why do people self harm?	http://www.adullam-ministries.org.uk/site/self-harm/why-do-people-self-harm/
4	Related struggles	http://www.adullam-ministries.org.uk/site/self-harm/related-struggles/
5	Getting help	http://www.adullam-ministries.org.uk/site/self-harmers/getting-help/
6	Alternatives	http://www.adullam-ministries.org.uk/site/self-harmers/alternatives/
7	Survivors’ stories	http://www.adullam-ministries.org.uk/site/self-harmers/survivors-stories/
8	How to respond	http://www.adullam-ministries.org.uk/site/carers/how-to-respond/

47. Scottish Association for Mental Health

Link: https://www.samh.org.uk/

Date searched: 21/8/19

Search strategy:

- Website browsed (2 hits)
- No search bar identified

Total hits = 2

Results which met eligibility criteria for full-text inclusion:

#	Webpage Name	Link
1	Self harm information	https://www.samh.org.uk/about-mental-health/mental-health-problems/self-harm
2	Understanding self harm	https://www.samh.org.uk/documents/SAMH_Understanding_Self_Harm.pdf

48. Action Mental Health Northern Ireland

Link: https://www.amh.org.uk/

Date searched: 21/8/19

Search strategy:

- Website browsed
- Individually searched “self harm”, “self injury”, “parasuicide”, “self injurious behaviour”, “deliberate self harm”, “self cutting” and “self poisoning” in main search bar

Total hits 5, of which 0 were duplicates

No results met eligibility criteria for full-text screening

49. The King's Fund

Link: https://www.kingsfund.org.uk/

Date searched: 20/9/19

Search strategy:

- Website browsed
- Individually searched "self harm", "self injury", "parasuicide", "self injurious behaviour", "deliberate self harm", "self cutting" and "self poisoning" in main search bar

Total hits = 19, of which 1 was a duplicate

Results which met eligibility criteria for full-text inclusion:

#	Webpage Name	Link
1	2019 GKS IMPACT Awards: Off the Record Youth Counselling Croydon	https://www.kingsfund.org.uk/audio-video/2019-gsk-impact-awards-off-the-record
2	Improving mental health: recognising the vital role of charities	https://www.kingsfund.org.uk/blog/2015/05/improving-mental-health-recognising-vital-role-charities
3	Claire Murdoch – mental health: from rhetoric to reality?	https://www.kingsfund.org.uk/audio-video/claire-murdoch-mental-health-rhetoric-reality
4	Christopher Hilton: Integrating physical health, mental health and social care	https://www.kingsfund.org.uk/audio-video/christopher-hilton-integrating-physical-mental-social-care
5	10 priorities for integrating physical and mental health	https://www.kingsfund.org.uk/publications/physical-and-mental-health/priorities-for-integrating
6	Charities win prestigious awards for improving health in the UK	https://www.kingsfund.org.uk/press/press-releases/charities-win-prestigious-awards-improving-health-uk
7	Young people's health charity steps forward to claim top accolade	https://www.kingsfund.org.uk/press/press-releases/young-peoples-health-charity-steps-forward-claim-top-accolade
8	Health charities win prestigious awards for life-changing words	https://www.kingsfund.org.uk/press/press-releases/health-charities-win-prestigious-awards-life-changing-work

Supplementary C. Results of the advanced Google search.

#	Title	Link	Met Criteria?	Reason for Exclusion
1	Self-harm - NHS	https://www.nhs.uk/conditions/self-harm/	No	Duplicate
2	Self-harm – Royal College of Psychiatrists	https://www.rcpsych.ac.uk/mental-health/problems-disorders/self-harm	No	Duplicate
3	Self-harm - Wikipedia	https://en.wikipedia.org/wiki/Self-harm	Yes	
4	Self-harm – Mind, the mental health charity	https://www.mind.org.uk/information-support/types-of-mental-health-problems/self-harm/#.XYyEB1VKiUk	No	Duplicate
5	Self-harm - YoungMinds	https://youngminds.org.uk/find-help/feelings-and-symptoms/self-harm/	No	Duplicate
6	The truth about self-harm – Mental Health Foundation	https://www.mentalhealth.org.uk/publications/truth-about-self-harm	Yes	
7	Self-harm – Mental Health Foundation	https://www.mentalhealth.org.uk/a-to-z/s/self-harm	Yes	

8	Self-injurious behaviour – National Autistic Society	https://www.autism.org.uk/about/behaviour/challenging-behaviour/self-injury.aspx	Yes	
9	Home – SelfHarmUK	https://www.selfharm.co.uk/	No	Duplicate
10	Overview – Self-harm – Quality standards - NICE	https://www.nice.org.uk/guidance/qs34	No	Duplicate
11	Self-harm - Childline	https://www.childline.org.uk/info-advice/your-feelings/self-harm/	No	Duplicate
12	Self-harm overview – NICE pathways	https://pathways.nice.org.uk/pathways/self-harm	No	Duplicate
13	Self-harm – NHS inform	https://www.nhsinform.scot/illnesses-and-conditions/mental-health/self-harm	Yes	
14	What is self-harm – MQ: Transforming Mental Health	https://www.mqmentalhealth.org/articles/self-harm	Yes	
15	Cutting and self-harm – HelpGuide.org	https://www.helpguide.org/articles/anxiety/cutting-and-self-harm.htm	No	Outside UK
16	Cutting & self-harm: Warning Signs and Treatment	https://www.webmd.com/mental-health/features/cutting-self-harm-signs-treatment#1	No	Outside UK
17	Self-harm – Rethink Mental Illness, the mental health charity	https://www.rethink.org/advice-and-information/about-mental-illness/learn-more-about-symptoms/self-harm/	No	Duplicate
18	Self-injury/cutting – Symptoms and causes – Mayo Clinic	https://www.mayoclinic.org/diseases-conditions/self-injury/symptoms-causes/syc-20350950	No	Outside UK
19	The NSHN Forum	http://www.nshn.co.uk/	No	Forum
20	Self-harm: MedlinePlus	https://medlineplus.gov/selfharm.html	No	Outside UK
21	Deliberate self-harm in adolescents - NCBI	https://www.ncbi.nlm.nih.gov/pmc/articles/PMC4469847/	No	Empirical study
22	The epidemiology of self-poisoning in the UK – NCBI	https://www.ncbi.nlm.nih.gov/pmc/articles/PMC1884308/	No	Empirical study
23	NHS figures show 'shocking' rise in self-harm among young people	https://www.theguardian.com/society/2016/oct/23/nhs-figures-show-shocking-rise-self-harm-young-people	Yes	
24	Self-harm – Psychology Today UK	https://www.psychologytoday.com/gb/basics/self-harm	Yes	
25	Self-harm - NSPCC	https://www.nspcc.org.uk/preventing-abuse/keeping-children-safe/self-harm/	Yes	
26	Self-harm - LifeLine	https://www.lifeline.org.au/get-help/topics/self-harm	No	Outside UK
27	Self-harm – NAMI: National Alliance on Mental Illness	https://www.nami.org/learn-more/mental-health-conditions/related-conditions/self-harm	No	Outside UK
28	Self Injury Support Ltd	https://www.selfinjurysupport.org.uk/	No	Duplicate
29	Self-harm – Time to Change	https://www.time-to-change.org.uk/category/blog/self-harm	Yes	
30	Self-Injurious Behaviour: Summary. About Behaviour.	https://www.challengingbehaviour.org.uk/understanding-behaviour/self-injurious-behaviour-sheet.html	Yes	
31	What self-injury is - LifeSIGNS	http://www.lifesigns.org.uk/what/	Yes	
32	Suicide and parasuicide – a comparison	https://www.gpnotebook.co.uk/simplepage.cfm?ID=-328531967	Yes	

33	Trends in self-poisoning and psychotropic drug use in people aged 5-19 years	https://bmjopen.bmj.com/content/9/2/e026001	No	Empirical study
34	BBC Radio 1 – BBC Advice – Self-harm	https://www.bbc.co.uk/programmes/articles/1LJd4YvhLTnLRH7WgF8pypJ/self-harm	No	Content unavailable
35	Self-harm and Suicide Prevention Competency Framework	https://www.ucl.ac.uk/pals/research/clinical-educational-and-health-psychology/research-groups/core/competence-frameworks/self	No	Duplicate
36	What is self-harm? Parents' guide – Internet Matters	https://www.internetmatters.org/issues/self-harm/	Yes	
37	SCIE Research briefing 16: Deliberate self-harm	https://www.scie.org.uk/publications/briefings/briefing16/	Yes	
38	Self-injury (Cutting, Self-Harm or Self-Mutilation	https://www.mhanational.org/conditions/self-injury-cutting-self-harm-or-self-mutilation	No	Outside UK
39	Self-harm – The Scout Association	https://members.scouts.org.uk/supportresources/2444/selfharm?cat=299,303&moduleID=10	Yes	
40	Self-harm – Definition of Self-Harm by Merriam-Webster	https://www.merriam-webster.com/dictionary/self-harm	No	Outside UK
41	The Cornell Research Programme on Self-Injury and Recovery	http://www.selfinjury.bctr.cornell.edu/	No	Outside UK
42	Self-harm – latest news, breaking stories and comment – The Independent	https://www.independent.co.uk/topic/self-harm	Yes	
43	Deliberate self-harm in the UK armed forced: index – GOV.UK	https://www.gov.uk/government/collections/deliberate-self-harm-in-the-uk-armed-forces-index	Yes	
44	Adolescent self-harm – Association for Young People's Health	http://www.youngpeopleshealth.org.uk/wp-content/uploads/2015/07/316_RU13-Self-harm-summary.pdf	Yes	
45	Self-harm Service – SLaM National Services	https://www.national.slam.nhs.uk/services/adult-services/selfharm/	Yes	
46	Guidance for people working with young people at risk from self-harm or suicide published	https://www.glasgow.gov.uk/article/19042/Guidance-for-people-working-with-young-people-at-risk-from-self-harm-or-suicide-published	Yes	
47	Encyclopaedia: self-harm – NHS Direct Wales	https://www.nhsdirect.wales.nhs.uk/Encyclopaedia/s/article/selfharm/	Yes	
48	Counselling for Self-Harm – Counselling Directory	https://www.counselling-directory.org.uk/self-harm.html	Yes	
49	Self-harm in girls and young women rising at 'alarming' rate	https://news.sky.com/story/self-harm-in-girls-and-young-women-rising-at-alarming-rate-11735074	Yes	

50	Self-Harm Project Aberdeen – Penumbra	http://www.penumbra.org.uk/service-locations/north-area-services/aberdeen/self-harm/	Yes	
51	Self-Harm – University of Plymouth	https://www.plymouth.ac.uk/student-life/services/student-services/shine/self-harm	Yes	
52	Self-harm – St Bede's School	https://www.st-bedes.surrey.sch.uk/1167/self-harm	Yes	
53	Self-Injury – Autism Research Institute	https://www.autism.org/self-injury/	No	Outside UK
54	Suicide and Self-Harm: Cumbria County School	http//www.cumbrialscb.com/LSCB/professionals/ssh.asp	Yes	
55	Self-harm in children and young people – information and advice for parents and carers	https://infolink.suffolk.gov.uk/kb5/suffolk/infolink/advice.page?id=0pYooMSGlxM	Yes	
56	Factsheet: Self-Injurious Behaviour	file:///C:/Users/meddrom/Downloads/Self%20Injurious%20Behaviour%2008%20(1).pdf)	No	Content unavailable
57	If you want to self-harm - Samaritans	https://www.samaritans.org/how-we-can-help/support-and-information/if-youre-having-difficult-time/if-you-want-self-harm/	No	Duplicate
58	Self-harm in England and Wales: an epidemiological study of prevalence, risk factors, clustering and subsequent suicide	https://www.thelancet.com/journals/lancet/article/PIIS0140-6736(13)62118-2/fulltext	No	Empirical study
59	Self-harm – Student home, University of York	https://www.york.ac.uk/students/health/advice/self-harm/	Yes	
60	Self-harm – Nightingale Hospital London	https://www.nightingalehospital.co.uk/self-harm/	Yes	
61	Support Organisations – Self-Harm – Channel 4	https://www.channel4.com/4viewers/help/self-harm	Yes	
62	Automutilation – an overview – ScienceDirect Topics	https://www.sciencedirect.com/topics/neuroscience/automutilation	No	Empirical study
63	NSHN – What is Self-harm?	http://www.nshn.co.uk/whatis.html	Yes	
64	Self-Harm – CAMHS	http://www.blackcountryminds.com/help-advice/self-harm/	Yes	
65	Intentional self-harm in adolescence	https://assets.publishing.service.gov.uk/government/uploads/system/uploads/attachment_data/file/621068/Health_behaviour_in_school_age_children_self-harm.pdf	Yes	
66	Self-harm in older adults: a forgotten group?	https://www.nationalelfservice.net/mental-health/self-harm/self-harm-in-older-adults/	Yes	
67	Self-Injury Information, Resources & Support - HealthyPlace	https://www.healthyplace.com/abuse/self-injury/self-injury-homepage	No	Outside UK

68	What is self-harm? – Self-harm – ReachOut Australia	https://au.reachout.com/articles/what-is-self-harm	No	Outside UK
69	Glorifying self-harm in adolescence – Twitter help centre	https://help.twitter.com/en/rules-and-policies/glorifying-self-harm	No	Social media
70	BACP CPD hub – Self-harm	https://www.bacp.co.uk/cpd/cpd-hub/self-harm/	No	Content unavailable
71	Self-harm – Stem4	https://stem4.org.uk/self-harm/	Yes	
72	Talk to me 2: suicide and self-harm prevention strategy for…	https://socialcare.wales/research-and-data/research-on-care-finder	No	Content unavailable
73	Condition – Self harm – Sussex Partnership NHS Foundation Trust	https://www.sussexpartnership.nhs.uk/condition-self-harm	Yes	
74	Self-harm and Risky Behaviour – MindEd hub	https://www.minded.org.uk/Component/Details/445676	No	Content unavailable
75	Child and Adolescent Self-Harm in Europe – A study	http://ec.europa.eu/justice/grants/results/daphne-toolkit/content/child-and-adolescent-self-harm-europe-study_en	No	Empirical study
76	Dudley and Walsall – Mental Health partnerhip >> Self-Harm	http://www.dwmh.nhs.uk/service-users-and-carers/self-harm/ (	Yes	
77	Nonsuicidal self-harm among community adolescents: understanding the “whats” and “whys” of self-harm	https://link.springer.com/article/10.1007/s10964-005-7262-z	No	Empirical study
78	Self-harm - erscb	http://www.erscb.org.uk/professionals-and-volunteers/self-harm/	Yes	
79	Self-harm – Avon and Wiltshire Mental Health Partnerships NHS Trust	http://www.awp.nhs.uk/advice-support/conditions/self-harm/	Yes	
80	Self-Harm - SAMH	https://www.samh.org.uk/documents/UnderstandingSelfHarm.pdf	No	Duplicate
81	Using data to inform suicide and self-harm prevention - StatsLife	https://www.statslife.org.uk/events/eventdetail/1346/-/using-data-to-inform-suicide-and-self-harm-prevention	No	Content unavailable
82	What is self-harm? – Rape Crisis England and Wales	https://rapecrisis.org.uk/get-help/looking-for-tools-to-help-you-cope/self-harm/what-is-self-harm/	Yes	
83	Self harm: Student Services: University of Dundee	https://www.dundee.ac.uk/student-services/counselling/self-help/self-harm/	Yes	
84	Suicide and self-harm in Britain – NatCen Social Research	http://natcen.ac.uk/our-research/research/suicide-and-self-harm-in-britain-researching-risk-and-resilience/	Yes	
85	Self-harm and suicide prevention frameworks – National Suicide Prevention Alliance	https://www.nspa.org.uk/resources/self-harm-and-suicide-prevention-frameworks/	No	Duplicate

86	Health and wellbeing self-harm	http://www.nwbh.nhs.uk/healthandwellbeing/Pages/Self-harm.aspx	Yes	
87	Self-harm - CAMHS	https://www.camhsnorthderbyshire.nhs.uk/self-harm	Yes	
88	About Self Harm – Coventry and Warwickshire Mind	https://cwmind.org.uk/about-self-harm/	Yes	
89	Self-harm Masterclass 'Therapeutic Assessment for adolescents with self-harm'	https://www.acamh.org/event/self-harm-0619/	Yes	
90	Self harm – The Tavistock and Portman NHS Foundation Trust	https://tavistockandportman.nhs.uk/care-and-treatment/conditions/self-harm/	Yes	
91	Self-Injury Outreach and Support	http://sioutreach.org/	No	Outside UK
92	Instagram to ban all graphic self-harm images from platform	https://www.telegraph.co.uk/news/2019/02/07/instagram-ban-graphic-self-harm-images-platform/	Yes	
93	Self Harm – The Corner Dundee – Health and Info for Young People	https://www.thecorner.co.uk/88_SelfHarm.html	Yes	
94	A History of Self-Harm in Britain – A Genealogy of Cutting and Overdosing	https://www.palgrave.com/gp/book/9781137529619	No	Empirical study
95	Self-Injury – Instagram Help Centre	https://help.instagram.com/553490068054878	No	Social media
96	Self Injury Support – Sheffield Mental Health Guide	https://www.sheffieldmentalhealth.co.uk/support/self-injury-support/	Yes	
97	From self-harm to self-belief	https://www.hgi.org.uk/resources/delve-our-extensive-library/resources-and-techniques/self-harm-self-belief	Yes	
98	Self-harm – The Mighty	https://themighty.com/topic/self-harm/	No	Social media
99	Self-Harm Intervention Programme (SHIP)	https://www.inspirewellbeing.org/mentalhealth/services/self-harm-intervention-programme-ship	Yes	
100	Young people use self-harm to share emotions	https://www.sv.uio.no/psi/english/research/news-and-events/news/young-people-use-self-harm-to-share-feelings.html	Yes	
